# Potential Capacity of Aptamers to Trigger Immune Activation in Human Blood

**DOI:** 10.1371/journal.pone.0068810

**Published:** 2013-07-23

**Authors:** Meltem Avci-Adali, Heidrun Steinle, Tatjana Michel, Christian Schlensak, Hans P. Wendel

**Affiliations:** Department of Thoracic, Cardiac, and Vascular Surgery, University Hospital Tuebingen, Tuebingen, Germany; University of Hawaii Cancer Center, United States of America

## Abstract

Target specific short single-stranded DNA (ssDNA) molecules, called aptamers, are auspicious ligands for numerous in vivo applications. However, aptamers are synthetic molecules, which might be recognized by the immune cells in vivo and induce an activation of the innate immune system. Thus, immune activation potential of synthetic ssDNA oligonucleotides (ODNs) was determined using a well established closed-loop circulation model. Fresh human blood was incubated at 37°C for 2 or 4 hours with ssDNA ODNs (SB_ODN) or CpG ODN as positive control. Transcriptional changes were determined by microarray analyses. Blood samples containing SB_ODN demonstrated after 4 hours a significant regulation of 295 transcripts. Amongst others, CCL8, CXCL10, CCL7 and CXCL11 were highest regulated genes. Gene Ontology terms and KEGG pathway analyses exhibited that the differentially expressed genes belong to the transcripts that are regulated during an immune and inflammatory response, and were overrepresented in TLR signaling pathway. This study shows for the first time the potential of aptamers to activate immune system after systemic application into the human blood. Thus, we highly recommend performing of these preclinical tests with potential aptamer-based therapeutics.

## Introduction

Aptamers are single-stranded DNA (ssDNA) or RNA oligonucleotides with a length of generally less than 100 bases. They can fold into well-defined three dimensional structures and bind their targets with high affinity and specificity. Using the combinatorial chemistry process SELEX (Systematic Evolution of Ligands by Exponential enrichment), aptamers can be selected from a large combinatorial pool of sequences against a wide variety of target molecules ranging from small molecules (amino acids, antibiotics), peptides, proteins to even whole living cells [1]. Hitherto, several aptamers have been developed for in vivo applications against biomedical relevant targets, such as coagulation factors [2], growth factors or cytokines [3], inflammation markers [4], stem [5] or cancer cells [6].

However, aptamers are synthetic nucleic acids, thus it is possible that they can be recognized by the innate immune system if they are used in vivo. Invading pathogens are recognized by human innate immune system via germline-encoded-pattern-recognition receptors (PRRs) which detect components of foreign pathogens referred to as pathogen-associated molecular patterns (PAMPs). Cells express several classes of PRRs, such as the Toll-like receptors (TLRs) and retinoic acid-inducible gene (RIG)-I-like receptors (RLRs) that recognize nucleic acids derived from viruses and bacteria and induce immune responses. TLR family belongs to one of the best-characterized signal-generating membrane bound receptors among the PRRs and comprises a set of nucleic-acid sensing TLRs, namely TLR3, TLR7, TLR8, and TLR9. Double-stranded RNA (dsRNA) is recognized by TLR3. TLR7 and TLR8 recognize single-stranded RNA (ssRNA) and TLR9 recognizes unmethylated 2′-deoxyribo (cytidine-phosphate-guanosine, CpG) DNA motifs that are commonly present in viruses and bacteria but are rare in mammalian cells. The RLRs are localized in the cytoplasm and recognize different RNA viruses by detecting both dsRNAs and ssRNAs that contain a 5′triphosphate end [7].

Previously, it was believed that only TLR9, which is expressed in humans in B cells as well as in plasmacytoid dendritic cells (pDCs), can detect DNA and leads to IFNα induction via myeloid differentiation primary response gene 88 (MYD88) and IKKα. However, in 2006, Ishii and colleagues demonstrated that immune stimulatory activity of DNA was not affected in many cells lacking TLR9 [8]. Since then at least six intracellular receptors have been identified that are sensing DNA. These are RNA polymerase III (Pol III) [9], DAI (DNA-dependent activator of interferon-regulatory factors), also called Z-DNA binding protein 1 (ZBP1) [10], Lrrfip1 (leucine-rich repeat (in Flightless I) interacting protein-1) [11], AIM2 (absent in melanoma 2) [12], DExD/H box helicases (DHX9 and DHX36) [13], and IFI16 (interferon inducible protein) [14]. Today, it is known that immune responses to DNA are not restricted to type I IFN inducing pathways. Cytosolic DNA from invading bacteria and viruses also activates caspase-1-dependent maturation of the cytokines IL-1b and IL-18.

Aptamers are synthetic oligonucleotides (ODNs) and can comprise one or more CpG motifs or other immunostimulatory sequences, which might induce an immune activation. Especially, when aptamers are used in vivo, the immune stimulatory potential of aptamers should be clearly examined. Hitherto, the immunostimulatory potential of DNA aptamers was not examined in any study. To our knowledge, this study is the first study which investigates the immune activation potential of aptamers in fresh human whole blood. For the investigation, human peripheral blood was incubated with a synthetic random DNA oligonucleotide library comprising of approximately 10^15^ different ssDNA molecules as aptamer candidates or a CpG oligonucleotide (CpG_ODN) as positive control, which is already known to induce immune activation. Subsequently, microarray analyses were performed to determine gene expression changes in whole human peripheral blood.

## Materials and Methods

### Ethics statement

The Ethics Committee of the University of Tübingen approved the blood sampling procedures and all subjects gave written informed consent.

### ssDNA oligonucleotides

For the incubation with human peripheral blood, a synthetic ssDNA oligonucleotide start library (SB_ODN) with a length of 66 nucleotides and a phosphodiester backbone, which is used in the combinatorial chemistry process SELEX (Systematic Evolution of Ligands by Exponential Enrichment) to select target binding aptamers, 5′-GCCTGTTGTGAGCCTCCTAAC-N25-CATGCTTATTCTTGTCTCCC-3′ and a CpG oligonucleotide M362 5′-TCGTCGTCGTTCGAACGACGTTGAT-3′ (CpG_ODN) with a length of 25 nucleotides and a phosphorothioate backbone were ordered from Ella Biotech (Martinsried, Germany) in HPLC-grade. Lyophilized oligonucleotides were reconstituted with sterile water for injection (Ampuwa®, Fresenius Kabi, Bad Homburg, Germany). The SB_ODN contained approximately 10^15^ different oligonucleotides consisting of a centrally randomized region of 25 nucleotides flanked by two fixed regions. To amplify the SB_ODN during the quantitative real-time PCR, a sense primer 5′-GCCTGTTGTGAGCCTCCTAAC-3′ and an antisense primer 5′-GGGAGACAAGAATAAGCATG-3′ were also obtained from Ella Biotech (Martinsried, Germany) in HPLC-grade.

### Determination of endotoxin and pyrogen levels in ordered oligonucleotides

Contaminating endotoxins/pyrogens in the ordered oligonucleotides may induce artificial immune response. Thus, synthetic ODNs, which are being tested for their ability to induce immune response, have to be tested for endotoxins/pyrogens in order to allow clear interpretation of their immunostimulatory effects. To exclude an activation of blood cells by potentially existing endotoxins/pyrogens, the endotoxin/pyrogen levels in the ordered oligonucleotides were examined using two different tests, namely Limulus amebocyte lysate (LAL) assay and monocyte activation test (MAT).

#### Limulus amebocyte lysate (LAL) assay

LAL reacts with bacterial endotoxin, like lipopolysaccharide (LPS) which is a membrane component of Gram-negative bacteria, and induces coagulation. A chromogenic LAL portable endotoxin detection system, Endosafe® Portable Test System (PTS™, Charles River Laboratories, Wilmington, US), and LAL test cartridges with a sensitivity of 0.5–0.005 EU (endotoxin units)/ml were used to detect and quantify bacterial endotoxin levels in the ordered ssDNA oligonucleotide solutions. Measurements were performed according to manufacturer's instructions.

#### Monocyte activation test (MAT) using fresh human whole blood

MAT was performed using the commercially available Biotest PyroDetect System (Biotest AG, Dreieich, Germany). The test uses an innate immune defense reaction of the human blood. Monocytes present in the human whole blood respond to pyrogens by producing cytokines, such as interleukin 1β (IL-1β) which is detected in an immunological assay (ELISA) involving specific antibodies and an enzymatic color reaction.

Blood from healthy volunteers (n = 4) was collected using syringes (Multifly®, Sarstedt, Nümbrecht, Germany) connected to 9 ml lithium heparin tubes (S-Monovette®, Sarstedt, Nümbrecht, Germany). Subsequently, collected blood was pooled and 100 µl was used per assay. The examinations were performed according to manufacturer's instructions using the quantitative test method. Briefly, 100 µl of endotoxin controls, sterile water as negative control, or ODN samples (SB_ODN or CpG_ODN) with or without addition of 0.5 EU/ml external endotoxin (spikes), was added to 900 µl of 0.9% NaCl solution (Fresenius Kabi, Bad Homburg, Germany). All samples were then mixed with 100 µl of pooled whole blood and incubated in an incubator for 20 hours at 37°C in a humidified atmosphere containing 5% CO_2_. The samples were then mixed and centrifuged for 5 min at 400 g. The response to pyrogenic substances is determined by measurement of the IL-1β molecules present in the culture supernatant using IL-1β ELISA.

### Serum Stability of SB_ODN

To determine the stability of the used oligonucleotides against nucleases in human blood, a serum stability test was performed. For this purpose, 10 µg SB_ODN or CpG_ODN was added to 0.5 ml of fresh serum (n = 3) and incubated at 37°C. At 3 time points (0, 2, and 4 h), 50 µl samples were collected. Immediately following collection, each sample was shock frozen in liquid nitrogen and stored until examination at −80°C.

Oligonucleotides in the samples were purified from serum proteins by phenol/chloroform/isoamylalcohol extraction and ethanol precipitation, with subsequent rehydration in 50 µl RNAse- and DNase-free water. Samples (5 µl) were run on a 10% denaturing urea-polyacrylamide gel and stained with GelRed (Biotium Inc, Hayward, USA). Additionally to the denaturing polyacrylamide gel electrophoresis, the amount of SB_ODN in the samples was determined with a quantitative real time PCR (qPCR) assay. A standard curve of SB_ODN from 5 pg to 0.02 pg was used to determine the amount in the samples. Using iQ™ SYBR Green Supermix (Bio-Rad, Munich, Germany) and 400 nM sense and antisense primer, the quantitative real time detection of SB_ODN was performed. The qPCR reactions were run in triplicate in an iCycler iQ Real-Time PCR Detection System (Bio-Rad, Munich, Germany). Initial DNA denaturation was performed at 95°C for 3 min, followed by 30 cycles of denaturation at 95°C for 45 s, annealing at 58°C for 20 s, extension at 72°C for 20 s and final extension at 72°C for 5 min. The amount of SB_ODN in serum samples without incubation was set at 100% and the results are presented relative to control SB_ODN levels. PCR assays were performed in triplicate.

### Blood sampling for the incubation of oligonucleotides in the closed-loop model

A total volume of 90 ml blood was collected from the antecubital vein of non-medicated, healthy volunteers (two female donors [26 and 28 years] and a 39 years old male donor) (n = 3). For all donors the following exclusion criteria were imperative: smoking, drug taking (aspirin, antiphlogistics, antiallergics, etc.), pregnancy, oral contraceptives. The blood was anticoagulated with 3 IU (International Units)/ml unfractionated sodium heparin (Ratiopharm, Ulm, Germany) to avoid excessive coagulation activation. To evaluate the influence of synthetic ssDNA oligonucleotides on the cells of circulating peripheral blood, the samples were divided into 7 groups (Table 1). Baseline value samples (Group I) were obtained after blood collection without rotating in the closed-loop model. Negative control samples (Group II and V) did not include oligonucleotides but rotated in the closed-loop model.

**Table 1:** Groups for microarray analyses.

Group	Treatment
**I**	0 h, without ssDNA
**II**	2 h, without ssDNA
**III**	2 h, with 10 µM CpG_ODN
**IV**	2 h, with 10 µM SB_ODN
**V**	4 h, without ssDNA
**VI**	4 h, with 10 µM CpG_ODN
**VII**	4 h, with 10 µM SB_ODN

### Incubation of human peripheral blood with ssDNA oligonucleotides in the closed-loop model

The incubation of ssDNA oligonucleotides with fresh human blood was performed in an in vitro closed-loop model (modified Chandler-Loop) [15]. This model allows the mimicry of the blood movement in a blood vessel and the study of blood cell activation under conditions of dynamic flow. Polyvinyl chloride (PVC) tubings with a length of 50 cm and covalently bonded heparin (Carmeda® bonded (CB) tubing, 1/4×1/16 inch, Medtronic, Minneapolis, US) were filled with 12 ml venous blood sample anticoagulated with 3 IU heparin/ml (Ratiopharm, Ulm, Germany) and then closed into a circuit using a piece of silicone tubing. Background coagulation activation was limited by the use of hemocompatible Carmeda® bioactive surface. The tubing loops were rotated vertically at 30 rpm in a water bath (37°C) for 2 and 4 h.

### Blood cell count

Cell count was measured in 100 µl blood samples before and after incubation in the closed-loop model using a fully automated hematology analyzer ABX Micros 60 (Horiba ABX, Montpellier, France).

### Detection of activation markers

Before and after incubation in the in vitro closed-loop, 1.4 ml of blood samples were filled in sodium citrate tubes (S-Monovette®, Sarstedt, Nümbrecht, Germany) and centrifuged immediately at 1800 g for 18 min at 20°C. The blood plasma of each sample was shock frozen in liquid nitrogen and stored at −80°C until further investigations. The plasma concentration of the coagulation marker thrombin-antithrombin-III complex (TAT) was determined using an Enzygnost® TAT micro enzyme-linked immunosorbent assay (Siemens Healthcare Diagnostics Products, Marburg, Germany). Polymorphonuclear (PMN) elastase release was measured using a commercially available ELISA kit (Milenia PMN-Elastase, Milenia Biotec GmbH, Gießen, Germany) to detect activation of polymorphonuclear neutrophils.

### Isolation of total RNA from blood samples

Total RNA was isolated from 10 ml whole blood using the RNeasy Midi Kit (Qiagen, Hilden, Germany) according to manufacturer's instructions. The isolation procedure included an additional incubation step with DNase I (RNase-Free DNase Set, Qiagen, Hilden, Germany) to ensure that the final product was devoid of genomic DNA. RNA concentrations were evaluated using a Nanodrop ND-1000 spectrophotometer (Nanodrop Technologies, Wilmington, USA). The quality of total RNA was assessed using an Agilent 2100 bioanalyzer (Agilent RNA6000 PicoChip) and the RNA Integrity Number (RIN) algorithm [16]. All RNA samples were of similar high quality with RIN scores of above 8.

### Microarray analysis

For expression profiling 100 ng of total RNA was linearly amplified and biotinylated using the GeneChip HT 3′IVT Express Kit (Affymetrix, Santa Clara, CA) according to the manufacturer's instructions. 15 µg of labeled and fragmented cRNA was hybridized onto Human Genome U219 Gene Chip® arrays (Affymetrix). Hybridization, washing, staining and scanning was performed automatically in a GeneTitan^TM^ instrument (Affymetrix). Scanned images were subjected to visual inspection to control for hybridization artifacts and proper grid alignment and analyzed with AGCC 3.0 (Affymetrix) to generate CEL files. The data have been deposited in NCBI's Gene Expression Omnibus and are accessible through GEO Series accession number GSE46676 (http://www.ncbi.nlm.nih.gov/geo/query/acc.cgi? Acc  =  GSE46676).

All subsequent data analysis steps were performed on the software platform R 2.12.0 and Bioconductor 2.10.0 [17]. Initially, the expression data from all chips were background corrected, quantile normalized and summarized with RMA (Robust Multichip Average) [18]. Due to the design of the experiment, two parameters (treatment and time) have an impact on gene expression, while the influence of interindividual differences from the three different donors has to be taken into account. So a combined factor from treatment and time was used to design a linear model which captures the influence on gene expression levels while using the donor as random variable. A non-specific filter based on overall variance was applied to remove non informative genes before the fitting of the linear models was performed. The coefficients describing the expression profiles of the remaining probe sets were calculated and the standard errors were moderated using an empirical Bayesian approach [19]. From the F statistic the resulting p-values were established and corrected for multiple testing with „Benjamini-Hochberg“ [20]. To attribute significant regulations to individual contrasts, a decision matrix was generated based on the function 'decide tests' within the limma package, where significant up- or downregulations are represented by values of 1 or -1, respectively. A transcript was considered as differentially expressed if the fold-change was greater than 1.5 and the Benjamini and Hochberg-corrected p-value was less than 0.05. The value of the contrast is given as M-value (M), which represents a log2-fold change in expression level. It is an indicator of the treatment effects on the expression level of a gene. Thus, an M-value of 0 represents equal expression, an M-value of 1 represents a two-fold, and an M-value of for example 2 implies a four-fold increase, whereas an M-value of -1 corresponds to a two-fold decrease in the mean expression. The lists of differentially regulated transcripts were analyzed for over-representation of gene ontology (GO) terms and KEGG (Kyoto encyclopaedia of genes and genomes) pathways, respectively, using hypergeometric test. Additionally, the lists were analyzed for gene-gene interactions using the Ingenuity Pathways Analysis (IPA version 9.0, Ingenuity Systems®, www.ingenuity.com).

### Real-time quantitative RT-PCR (qRT-PCR)

To confirm the differential regulation pattern of genes observed by the microarray analysis, 26 genes (listed in Table 2) were selected for confirmation analysis by custom RT^2^ Profiler PCR arrays (CAPH-10912, SABiosciences/Qiagen, Hilden, Germany). The selection of the genes was performed based on their gene expression values such as CXCL10, CCL7, CCL2, CXCL11 as highly up-regulated genes, based on down-regulated genes such as PTPN7 and F2RL1, or based on their immunological relevance such as IFNA5, IFNB1, CD83, CD40, TLR9, TLR3, TLR7, and TLR8. For this purpose, 1 µg of each RNA sample was reverse transcribed to cDNA using the RT^2^ PCR Array First Strand Kit (SABiosciences/Qiagen, Hilden, Germany) according to supplier's recommendations. Reaction volumes of 25 µl were prepared using RT^2^ SYBR Green Fluor qPCR Master Mix (SABiosciences/Qiagen, Hilden, Germany). Real-time quantitative RT-PCR (qRT-PCR) reactions were run in an iCycler iQ Real-Time PCR Detection System (Bio-Rad, Munich, Germany). Expression of the constitutively expressed gene GAPDH (glyceraldehyde 3-phosphate dehydrogenase) was used as an internal control. PCR amplification of cDNA was performed under following conditions: 10 min at 95°C for one cycle, followed by 40 cycles of 95°C for 15 s and 60°C for 60 s. Each PCR was performed in triplicate. All mRNA Ct values for each sample [Ct (sample)] were normalized to glyceraldehyde-3-phosphate dehydrogenase [Ct (GAPDH)] in the same sample and the results are shown relative to control mRNA levels.

**Table 2:** List of genes for validation of microarray data by custom RT^2^ Profiler PCR Array.

Gene	Official Full Name	NCBI Refseq#
**CXCL10**	Chemokine (C-X-C motif) ligand 10	NM_001565
**CCL7**	Chemokine (C-C motif) ligand 7	NM_006273
**CCL2**	Chemokine (C-C motif) ligand 2	NM_002982
**IL6**	Interleukin 6 (interferon, beta 2)	NM_000600
**CXCL11**	Chemokine (C-X-C motif) ligand 11	NM_005409
**TLR2**	Toll-like receptor 2	NM_003264
**ICAM1**	Intercellular adhesion molecule 1	NM_000201
**PLAU**	Plasminogen activator, urokinase	NM_002658
**SERPINB2**	Serpin peptidase inhibitor, clade B (ovalbumin), member 2	NM_002575
**CD83**	CD83 molecule	NM_004233
**CD40**	CD40 molecule, TNF receptor superfamily member 5	NM_001250
**CD80**	CD80 molecule	NM_005191
**IL4I1**	Interleukin 4 induced 1	NM_152899
**TNFSF15**	Tumor necrosis factor (ligand) superfamily, member 15	NM_005118
**IFNA5**	Interferon, alpha 5	NM_002169
**IFNB1**	Interferon, beta 1, fibroblast	NM_002176
**RGL1**	Ral guanine nucleotide dissociation stimulator-like 1	NM_015149
**CD22**	CD22 molecule	NM_001771
**PTPN7**	Protein tyrosine phosphatase, non-receptor type 7	NM_002832
**F2RL1**	Coagulation factor II (thrombin) receptor-like 1	NM_005242
**TLR1**	Toll-like receptor 1	NM_003263
**TLR5**	Toll-like receptor 5	NM_003268
**TLR8**	Toll-like receptor 8	NM_138636
**TLR9**	Toll-like receptor 9	NM_017442
**TLR3**	Toll-like receptor 3	NM_003265
**TLR7**	Toll-like receptor 7	NM_016562
**GAPDH**	Glyceraldehyde-3-phosphate dehydrogenase	NM_002046

### Statistical analysis

Statistical analysis of microarray data is described in detail in section microarray analysis. All other data are shown as means ± standard error of mean (SEM). Student's paired-sample t-test was performed for comparison of means between two groups. One-way analysis of variance (ANOVA) was performed to compare the means of more than two groups. All statistical tests were performed double-tailed, using the Origin software package (OriginPro 8, Northampton, USA). Differences of p<0.05 were considered significant.

## Results

### Determination of endotoxin and pyrogen levels in ordered oligonucleotides

#### Limulus amebocyte lysate (LAL) assay

Endotoxin levels (Table 3) in the ordered oligonucleotides for the incubation with human whole blood were examined using the Endosafe®-PTS^TM^. Simultaneously, test samples together with a known amount of endotoxin, called spike, were used to determine the ability of the sample to react with the dry LAL reagent and form chromogenic compound. According to the manufacturer of the test system, the spike recovery rate must be for a valid assay between 50 and 200%, which indicates no significant interference from the test sample.

**Table 3:** Detection of endotoxins in the ordered oligonucleotides using the Limulus amebocyte lysate (LAL) assay.

Sample	Endotoxin Value (EU/ml)	PTS™ Recovery Test (%)	Endotoxin Limit Value (EU/ml)
**SB_ODN**	0.014	73	0.25
**CpG_ODN**	<0.005	90	

Endotoxin value measured in the SB_ODN sample was 0.014 EU/ml and in the CpG_ODN sample was <0.005 EU/ml. These data clearly showed that the endotoxin values in both samples were far below the acceptable endotoxin limit of 0.25 EU/ml, which is specified by USP (United States Pharmacopeia) as endotoxin limit for sterile water for injection (WFI). Also the spike recovery rates of SB_ODN and CpG_ODN samples given by the Endosafe®-PTS^TM^ were within the acceptable range.

#### Monocyte activation test (MAT) using fresh human whole blood

LAL assay shows best sensitivity to endotoxins, however it is limited to pyrogens originating from Gram-negative bacteria. To allow a more realistic prediction of the pyrogenic activity in the oligonucleotide samples, we additionally used monocyte activation test (Table 4). The validity of the test was determined by recovery rate of an added endotoxin spike. Recovery rate with both oligonucleotide samples was within the acceptable range of 50–200%. The pyrogen level in the SB_ODN sample was 0.068 EU/ml and in the CpG_ODN sample was 0.139 EU/ml. Thus, the measurements clearly demonstrated that the detected pyrogen levels in both samples were far below the endotoxin limit of 0.25 EU/ml.

**Table 4:** Detection of pyrogen levels in the ordered oligonucleotides using monocyte activation test (MAT).

Sample	Pyrogen Value (EU/ml)	PyroDetect Recovery Test (%)	Pyrogen Limit Value (EU/ml)
**SB_ODN**	0.068	77.3	0.25
**CpG_ODN**	0.139	65.8	

### Serum Stability of SB_ODN

**Figure 1:**
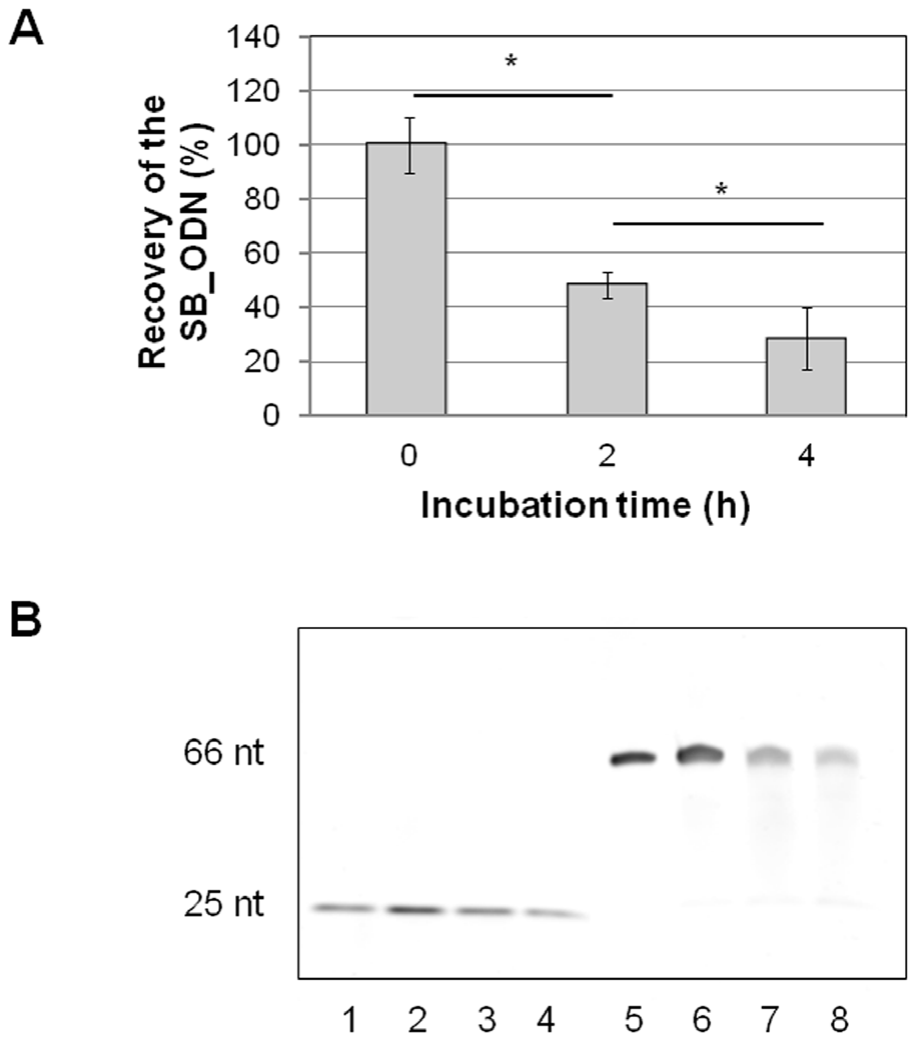
Serum stability of SB_ODN. A) Determination of the SB_ODN amount in serum samples without incubation (0 h), with 2 h or 4 h incubation at 37°C using qPCR. The results are presented as means ± SEM. Significance (p<0.05) is indicated by *.B) Analyses of the SB_ODN and CpG_ODN amount in serum samples using 10% denaturing urea-polyacrylamide gel electrophoresis. Lane 1: Positive Control: 200 ng CpG_ODN, Lane 2: CpG_ODN without incubation, Lane 3: CpG_ODN after 2 h serum incubation, Lane 4: CpG_ODN after 4 h serum incubation, Lane 5: Positive Control: 200 ng SB_ODN, Lane 6: SB_ODN without incubation, Lane 7: SB_ODN after 2 h serum incubation, Lane 8: SB_ODN after 4 h serum incubation.

The presence of primer regions in each ssDNA molecule of the SB_ODN enables the quantitative determination of the ssDNA amount after serum incubation by using SB_ODN specific primers and a standard curve with known SB_ODN amounts (Figure 1A). After 2 h incubation at 37°C in human serum, the amount of full length ssDNA molecules was reduced from 100% to 48%. And after 4 h, a further reduction of the SB_ODN amount to 29% was detected. These data demonstrated a reducing of the present SB_ODN amount by approximately 50% every 2 hours in human serum. Additionally to the qPCR measurements, denaturing urea-polyacrylamide gel electrophoresis was performed to visualize the amount of SB_ODN and CpG_ODN at 3 different incubation times (0, 2, and 4 h) with fresh human serum (Figure 1B). Visual examination of gels demonstrated consistent findings as with the qPCR. SB_ODN bands exhibited approximately a halving of the intensity every 2 hours. The samples with CpG_ODN also showed a reduction of the amount over time. Every 2 hours, the intensity of the CpG_ODN bands was approximately halved.

### Blood cell count

**Figure 2:**
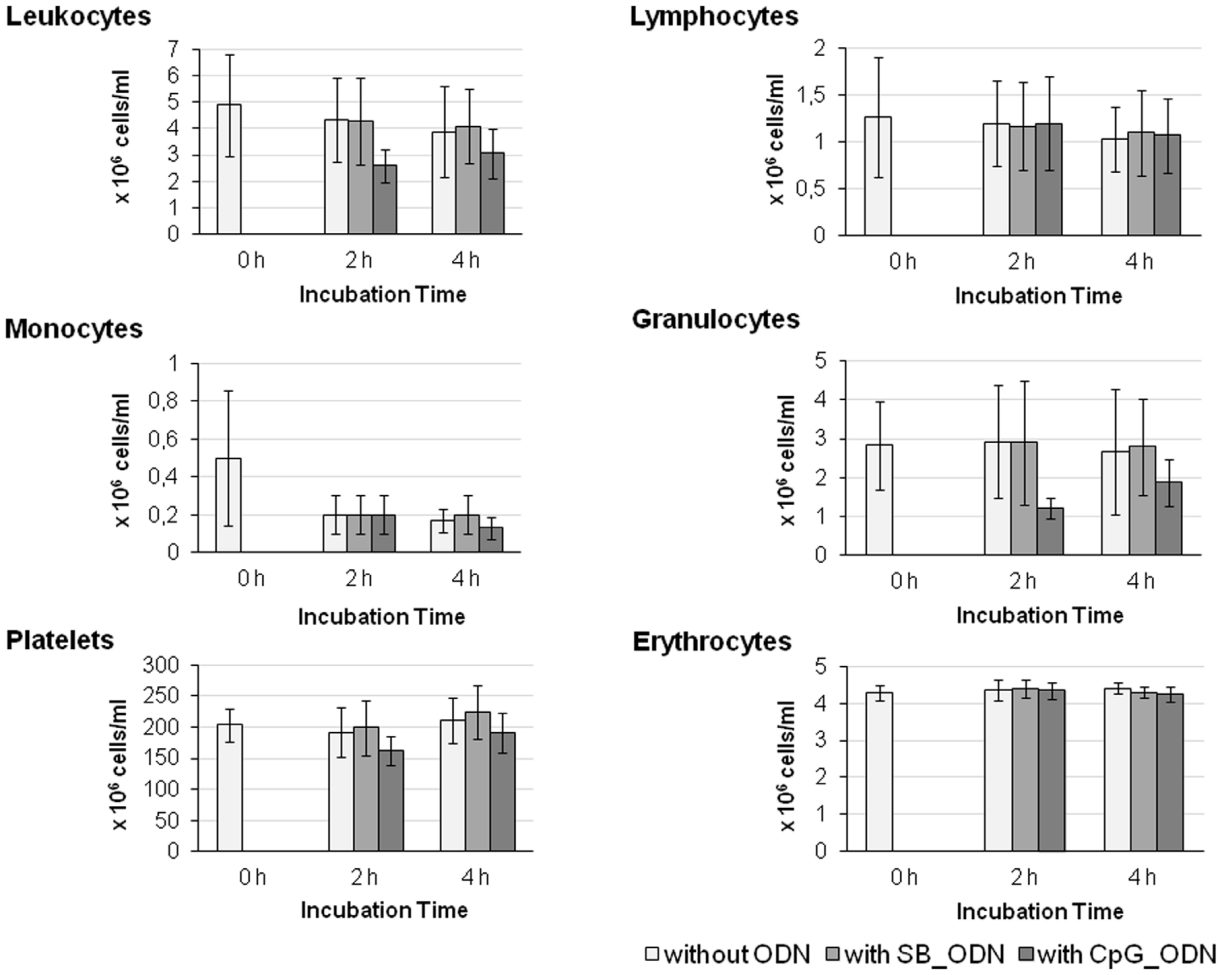
Analysis of blood cell counts. Quantity of leukocytes, lymphocytes, monocytes, granulocytes, platelets, and erythrocytes in the blood samples was measured before circulation in the closed-loop model and after the circulation without ODN, with SB_ODN, or CpG_ODN for 2 or 4 h. The results are presented as means ± SEM.

Cell counts of erythrocytes, platelets, granulocytes, monocytes, lymphocytes, and leucocytes were measured before and after 2 and 4 h incubation of human peripheral blood in the closed-loop model. Thereby, a possible cell-damaging effect of the blood circulation in the closed-loop model and addition of oligonucleotides to the blood was determined. As shown in Figure 2, after performing the in vitro closed-loop study, no significant cell count differences were identified compared to the initial cell number.

### Detection of activation markers

**Figure 3:**
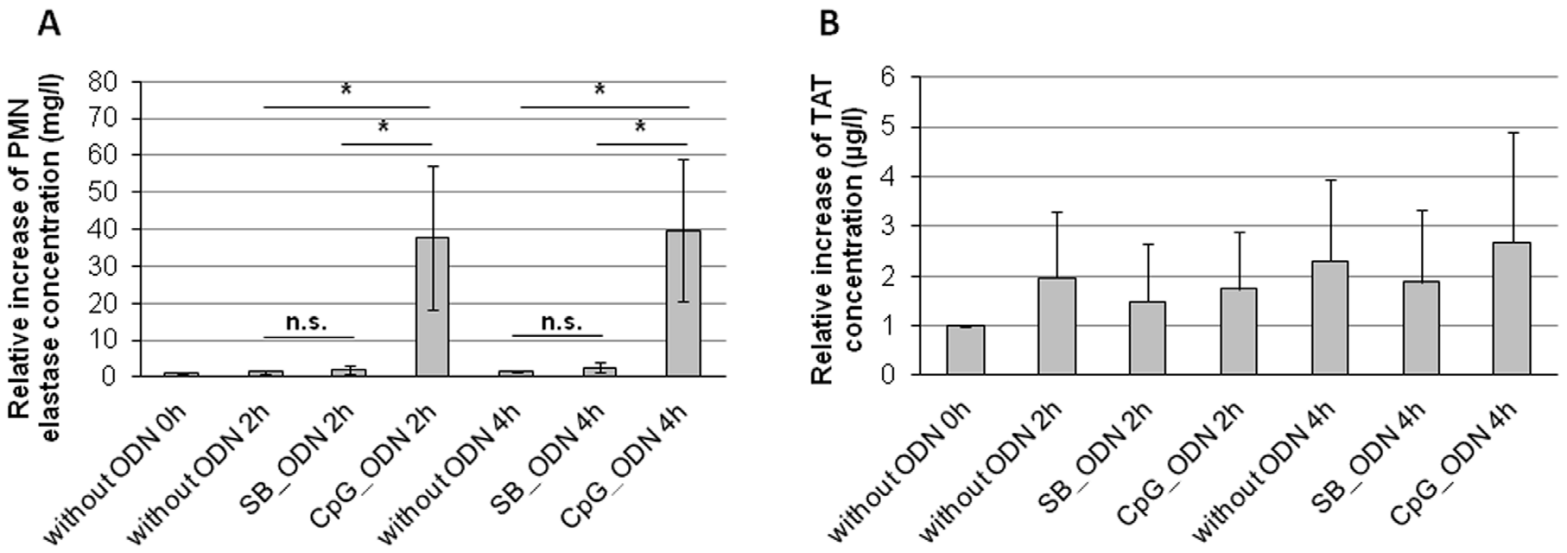
Measurement of activation markers in the blood samples after circulation in the closed-loop model and addition of ODNs. Determination of relative increase of A) PMN elastase concentration, B) TAT concentration (coagulation marker). The results are presented as means ± SEM. Significance (p<0.05) is indicated by *.

Release of polymorphonuclear (PMN) elastase was measured in blood samples before and after circulation in the in vitro closed-loop model and incubation with SB_ODN or CpG_ODN for 2 or 4 h. In comparison to the PMN elastase levels of blood samples directly after the collection, there was no significant increase due to circulation in the heparin coated closed-loops of the test system (Figure 3A). Also the incubation of blood with SB_ODN did not significantly change the PMN elastase levels compared to the blood samples without addition of ODNs. However, the addition of CpG_ODN and circulation for both 2 and 4 h led to a significant higher PMN elastase concentration in the blood samples than without ODN or SB_ODN incubation. PMN elastase level after 2 h incubation was approximately 19 times and after 4 h 26 times higher than in the control blood samples without ODN addition.

To examine coagulation activation, relative increase of TAT concentration in the blood samples was measured. As shown in Figure 3B, there was no significant increase of TAT concentration compared to the blood samples directly after the collection without circulation and after the circulation with or without addition of ODNs.

### Microarray analyses

#### Number of regulated transcripts

To investigate the expression alterations in human blood cells after 2 or 4 h incubation with 10 µM SB_ODN or CpG_ODN in an in vitro closed-loop model, gene expression analysis was performed using the Affymetrix Human Genome U219 array. Using this array, gene expression of 30,108 transcripts was measured per sample. In comparison to untreated samples, the absolute number and the relative percentage of differentially regulated transcripts in the samples incubated with oligonucleotides is shown in Table 5. After incubation of human blood for 2 or 4 h with SB_ODN, less than 1% of the examined transcripts were regulated. Relative percentage of regulated transcripts after 2 h incubation with SB_ODN was only 0.03%. However, CpG_ODN led to an explicitly higher number of regulated transcripts than SB_ODN. Namely, after incubation for 2 h, 21.19% of the examined transcripts and after 4 h incubation, 25.14% of the examined transcripts were regulated. Both CpG_ODN and SB_ODN incubation for 4 h resulted in higher number of regulated transcripts than after 2 h. SB_ODN incubation for 4 h led to the regulation of approximately 30 times more transcripts than 2 h incubation. Against it, CpG_ODN incubation for 4 h showed about 1.2 times more gene regulations than after 2 h.

**Table 5:** The absolute number and the relative percentage of regulated transcripts compared to untreated samples.

Comparison	Absolute Number of Regulated Transcripts	Relative Percentage of Regulated Transcripts [%]
**2h: SB_ODN vs. Untreated**	10	0.03
**4h: SB_ODN vs. Untreated**	295	0.98
**2h: CpG_ODN vs. Untreated**	6379	21.19
**4h: CpG_ODN vs. Untreated**	7570	25.14

#### Differentially expressed transcripts

To screen the gene list obtained by microarray analysis for oligonucleotide induced expression changes, we focused our interest on genes that showed the highest differential gene expression in the ODN incubated blood samples versus control group. Table 6 shows a partial list of these transcripts ranked according to greatest fold change in expression.

**Table 6:** List of top 10 significantly up-regulated gene transcripts in response to incubation of human whole blood with CpG_ODN or SB_ODN for 2 and 4 h.

Rank	Symbol	Gene Name	UniGene ID	M-value	Fold Change
CpG_ODN 2h					
**1**	*PLAU*	plasminogen activator, urokinase	Hs.77274	7.701	208.08
**2**	*CCL2*	chemokine (C-C motif) ligand 2	Hs.303649	7.522	183.81
**3**	*CCL7*	chemokine (C-C motif) ligand 7	Hs.251526	7.246	151.76
**4**	*PHACTR1*	phosphatase and actin regulator 1	Hs.436996	6.878	117.65
**5**	*PHLDA1*	pleckstrin homology-like domain, family A, member 1	Hs.602085	6.346	81.35
**6**	*CXCL2*	chemokine (C-X-C motif) ligand 2	Hs.75765	6.324	80.13
**7**	*PPARG*	peroxisome proliferator-activated receptor gamma	Hs.162646	6.046	66.07
**8**	*NRIP3*	nuclear receptor interacting protein 3	Hs.523467	6.043	65.95
**9**	*PPAP2B*	phosphatidic acid phosphatase type 2B	Hs.405156	5.994	63.73
**10**	*TNFSF15*	tumor necrosis factor (ligand) superfamily, member 15	Hs.241382	5.952	61.92

CpG_ODN 4h					
**1**	*CCL8*	chemokine (C-C motif) ligand 8	Hs.271387	9.089	544.64
**2**	*CXCL10*	chemokine (C-X-C motif) ligand 10	Hs.632586	8.685	411.45
**3**	*CCL7*	chemokine (C-C motif) ligand 7	Hs.251526	7.883	236.06
**4**	IFNA5*	interferon. alpha 5	Hs.37113	7.330	160.94
**5**	*CCL2*	chemokine (C-C motif) ligand 2	Hs.303649	7.328	160.67
**6**	*IFNA10*	interferon. alpha 10	Hs.282275	7.295	157.09
**7**	*IFNA17*	interferon. alpha 17	Hs.282276	7.121	139.18
**8**	*IFNB1*	interferon. beta 1. fibroblast	Hs.93177	7.050	132.49
**9**	*CXCL11*	chemokine (C-X-C motif) ligand 11	Hs.632592	6.991	127.22
**10**	*IFNA1 /// IFNA13*	interferon. alpha 1 /// interferon. alpha 13	Hs.37026	6.808	112.02

SB_ODN 2h					
**1**	CD83	CD83 molecule	Hs.595133	1.725	3.30
**2**	PTPN7	protein tyrosine phosphatase, non-receptor type 7	Hs.402773	0.890	1.85
**3**	VPS53	vacuolar protein sorting 53 homolog (S. cerevisiae)	Hs.461819	0.718	1.65
**4**	NFKBID	nuclear factor of kappa light polypeptide gene enhancer in B-cells inhibitor, delta	Hs.466531	0.655	1.57
**5**	TOM1	target of myb1 (chicken)	Hs.474705	0.590	1.51
**6**	CAPN12	calpain 12	Hs.712632	0.589	1.50

SB_ODN 4h					
**1**	*CCL8*	chemokine (C-C motif) ligand 8	Hs.271387	5.117	34.71
**2**	*CXCL10*	chemokine (C-X-C motif) ligand 10	Hs.632586	4.802	27.89
**3**	*CCL7*	chemokine (C-C motif) ligand 7	Hs.251526	3.822	14.15
**4**	*CXCL11*	chemokine (C-X-C motif) ligand 11	Hs.632592	3.221	9.33
**5**	*SERPINB2*	serpin peptidase inhibitor, clade B (ovalbumin), member 2	Hs.594481	2.824	7.08
**6**	*PLAU*	plasminogen activator, urokinase	Hs.77274	2.712	6.55
**7**	*IL4I1*	interleukin 4 induced 1	–	2.686	6.43
**8**	*CCL3*	chemokine (C-C motif) ligand 3	Hs.514107	2.678	6.40
**9**	*RGL1*	ral guanine nucleotide dissociation stimulator-like 1	Hs.497148	2.591	6.02
**10**	*PHACTR1*	phosphatase and actin regulator 1	Hs.436996	2.407	5.30
M-value represents a log2-fold change in expression level					

Since very few transcripts were regulated in samples with SB_ODN incubation for 2 h, only 6 up-regulated transcripts are listed.

Only 6 transcripts were significantly up-regulated after 2 hours incubation with SB_ODN, from those CD83, which is a maturation marker for human dendritic cells, demonstrated the highest up-regulation with a fold change of 3.3 compared to untreated blood samples. Blood samples incubated with SB_ODN for 4 hours exhibited the highest up-regulation for the cytokines CCL8, CXCL10, CCL7, and CXCL11. Also in samples with CpG_ODN incubation for 4 h CCL8, CXCL10, and CCL7 belonged to the highest up-regulated transcripts. Further chemokines that were highly up-regulated were CXCL11, CCL2 and CXCL2 in CpG_ODN incubated samples and CCL3 in SB_ODN incubated samples. All these chemokines are involved in immune regulatory processes and inflammatory responses in which they control the chemotactic movement of immune cells, such as monocytes, polymorphonuclear leukocytes, T cells, or dendritic cells, to the sites of inflammation caused by tissue injury or infection.

The fold change of all significantly regulated transcripts was in CpG_ODN treated samples higher than in samples that were incubated with SB_ODN. For example, when comparing the fold change of the first 3 highly up-regulated transcripts after 4 h, CCL8 expression was approximately 16 times, CXCL10 expression was about 15 times, and the CCL7 expression was approximately 17 times lower in the SB_ODN incubated samples than in CpG_ODN treated samples.

As it is already known, CpG_ODN induced immune responses are based on the activation of immune cells expressing TLR9. The used CpG_ODN belongs to the class C CpG_ODN and it is able to activate B cells as well as pDCs. The characteristic features of pDCs upon activation of TLR9 are the production of large amounts of type I IFN (IFNA and IFNB). In this microarray studies, IFNA and IFNB belonged to the highly up-regulated transcripts after CpG_ODN incubation of human blood. In contrast, SB_ODN led to a slight up-regulation of IFNA and IFNB. However, it was statistically not significant.

The coagulation factor plasminogen activator inhibitor-2 (SERPINB2) is present in most cells, especially monocytes/macrophages and inactivates urokinase-type plasminogen activator (uPA) and tissue plasminogen activator (tPA), which are involved in fibronolysis. This factor showed after 4 hours incubation with SB_ODN about 7-fold increased transcript levels than in the untreated blood samples. However, simultaneously the urokinase-type plasminogen activator (PLAU, uPA) was also up-regulated. In CpG_ODN treated samples, an up-regulation of SERPINB2 and PLAU was determined respectively. After 4 h of incubation, approximately a fold change of 82 was detected for PLAU and a fold change of about 97 was detected for SERPINB2 (Table 7).

**Table 7:** Validation of microarray data using RT^2^ Profiler PCR Array.

		qRT-PCR		Microarray Analyses	
Gene	Treatment	p-Value	Up- or Down-Regulation	M	Fold Change
**CXCL10**	CpG_ODN_4h	<**0.01**	**16133.57**	**8.68**	**411.45**
	SB_ODN_4h	<**0.05**	**129.86**	**4.80**	**27.89**
**CCL7**	CpG_ODN_4h	<**0.05**	**2281.17**	**7.88**	**236.06**
	SB_ODN_4h	<**0.05**	**37.04**	**3.82**	**14.15**
**CCL2**	CpG_ODN_4h	<**0.01**	**1340.81**	**7.33**	**160.67**
	SB_ODN_4h	0.051	23.34	3.36	10.30
**IL6**	CpG_ODN_4h	<**0.01**	**315.17**	**5.14**	**35.17**
	SB_ODN_4h	0.054	26.60	**2.06**	**4.18**
**CXCL11**	CpG_ODN_4h	<**0.01**	**2964.01**	**6.99**	**127.22**
	SB_ODN_4h	<**0.05**	**54.44**	**3.22**	**9.33**
**TLR2**	CpG_ODN_4h	<**0.01**	**3.20**	**2.02**	**4.07**
	SB_ODN_4h	<**0.05**	**1.58**	**1.05**	**2.07**
**ICAM1**	CpG_ODN_4h	<**0.01**	**3.65**	**2.16**	**4.46**
	SB_ODN_4h	<**0.05**	**1.73**	**0.90**	**1.86**
**PLAU**	CpG_ODN_4h	<**0.01**	**104.77**	**6.35**	**81.47**
	SB_ODN_4h	<**0.05**	**6.70**	**2.71**	**6.55**
**SERPINB2**	CpG_ODN_4h	<**0.01**	**385.05**	**6.60**	**96.80**
	SB_ODN_4h	0.055	12.70	**2.82**	**7.08**
**CD83**	CpG_ODN_4h	<**0.01**	**19.25**	**3.93**	**15.27**
	SB_ODN_4h	<**0.05**	**4.13**	**2.11**	**4.32**
**CD40**	CpG_ODN_4h	<**0.01**	**12.22**	**3.36**	**10.30**
	SB_ODN_4h	<**0.05**	**2.70**	**1.52**	**2.87**
**CD80**	CpG_ODN_4h	<**0.01**	**19.55**	**4.25**	**19.01**
	SB_ODN_4h	<**0.05**	**4.70**	**1.73**	**3.32**
**IL4I1**	CpG_ODN_4h	<**0.01**	**48.50**	**5.62**	**49.13**
	SB_ODN_4h	<**0.05**	**6.20**	**2.69**	**6.43**
**TNFSF15**	CpG_ODN_4h	<**0.05**	**148.17**	**5.75**	**53.70**
	SB_ODN_4h	<**0.05**	**11.14**	2.05	4.15
**IFNA5**	CpG_ODN_4h	<**0.01**	**237.02**	**7.33**	**160.94**
	SB_ODN_4h	<**0.05**	**15.80**	2.69	6.46
**IFNB1**	CpG_ODN_4h	<**0.01**	**442.30**	**7.05**	**132.49**
	SB_ODN_4h	0.060	16.25	2.54	5.8
**RGL1**	CpG_ODN_4h	<**0.01**	**50.02**	**5.56**	**47.18**
	SB_ODN_4h	<**0.05**	**4.59**	**2.59**	**6.02**
**CD22**	CpG_ODN_4h	<**0.01**	**8.00**	**2.56**	**5.89**
	SB_ODN_4h	<**0.01**	**2.87**	**1.40**	**2.63**
**PTPN7**	CpG_ODN_4h	<**0.05**	**−1.39**	**−0.61**	**−1.52**
	SB_ODN_4h	0.339	1.08	0.34	1.27
**F2RL1**	CpG_ODN_4h	<**0.01**	**−6.65**	**−2.33**	**−5.01**
	SB_ODN_4h	0.573	**−**1.62	**−**0.54	**−**1.45
**TLR1**	CpG_ODN_4h	**<0.01**	**−5.66**	**−1.55**	**−2.92**
	SB_ODN_4h	0.500	**−**1.59	**−**0.35	**−**1.27
**TLR5**	CpG_ODN_4h	<**0.01**	**−4.56**	**−2.56**	**−5.91**
	SB_ODN_4h	0.473	**−**1.44	**−**0.34	**−**1.26
**TLR8**	CpG_ODN_4h	<**0.01**	**−6.50**	**−2.25**	**−4.77**
	SB_ODN_4h	<**0.05**	**−1.53**	**−**0.45	**−**1.37
**TLR9**	CpG_ODN_4h	<**0.01**	**−3.94**	n.d.	n.d.
	SB_ODN_4h	0.884	**−**1.05	n.d.	n.d.
**TLR3**	CpG_ODN_4h	<**0.01**	**3.48**	n.d.	n.d.
	SB_ODN_4h	0.235	1.12	n.d.	n.d.
**TLR7**	CpG_ODN_4h	0.212	**−**1.24	n.d.	n.d.
	SB_ODN_4h	0.394	**−**1.33	n.d.	n.d.

Comparative list of determined expression changes of selected transcripts after 4 h of blood incubation with SB_ODN or CpG_ODN using RT^2^ Profiler PCR Array and the microarray analysis.

Values, which are significantly up- or down-regulated, are written in bold. Not significantly regulated values are not highlighted. The abbreviation n.d. means not detected.

In SB_ODN treated samples for 4 h, an immunosuppressive enzyme, IL4I1 (Interleukin-4-induced gene 1), which can be expressed by B-cells and mononuclear phagocytes by various proinflammatory stimuli through the activation of the transcription factors NFκB and/or STAT1 and inhibits T cell proliferation, showed approximately 6.5-fold up-regulation compared to untreated samples. The expression of this enzyme was approximately 11-fold up-regulated in CpG_ODN treated samples after 2 h (Table S1 in File S1) and 50-fold up-regulated after 4 h compared to samples without ODNs (Table 7).

RGL1 (ral guanine nucleotide dissociation stimulator-like 1, also known as RGL or RalGDS-like 1) is a putative GEF (guanine nucleotide exchange factor) and a Ras effector, which regulates multiple processes, such as receptor endocytosis, cytoskeletal changes, and DNA synthesis. Ras-regulated signal pathways control diverse cell behaviors, such as cell migration, proliferation, differentiation, adhesion and apoptosis. SB_ODN stimulation of human blood for 4 h led to about 6-fold increase of RGL1. CpG_ODN stimulated samples also showed an up-regulation, more precisely, 9.38-fold after 2 h (Table S1 in File S1) and 47.18-fold after 4 h (Table 7).

PHACTR1 (phosphatase and actin regulator 1) controls the activities of protein phosphathase 1, which is a multifunctional enzyme that regulates cell progression, splicing of RNA, cell division, apoptosis, and protein synthesis. PHACTR1 also binds to cytoplasmic actin and controls F-actin remodeling. This enzyme belongs with a fold-change of 5.3 to the top ten highly up-regulated transcripts after SB_ODN stimulation for 4 h. An up-regulation was also detected in CpG_ODN treated samples, namely about 118-fold after 2 h and 47-fold after 4 h (data not shown).

### Verification of differential gene expression by real-time RT-PCR

Quantitative real-time RT-PCR was applied in order to approve the significance of transcriptional changes found by the microarray analyses. The mRNA expressions of 26 genes were examined using the custom RT^2^ Profiler PCR arrays (Table 7). The expression of the selected transcripts CXCL10, CCL7, CCL2, CXCL11, TLR2, ICAM1, PLAU, CD83, CD40, CD80, IL4I1, IFNB1, RGL1, CD22, PTPN7, F2RL1, TLR1, TLR5, and TLR7 demonstrated similar behavior as the detected expression changes in microarray analyses. In comparison to the fold-change determined by microarray analyses, some transcripts such as CXCL10, CCL7, CCL2, and CXCL11 demonstrated higher relative expression levels in RT^2^ Profiler PCR arrays. Although IL6 and SERPINB2 also demonstrated higher expression levels in SB_ODN incubated blood samples compared to samples without ODN, it was statistically not significant in RT^2^ Profiler PCR arrays. Presumably, due to the higher sensitivity of RT^2^ Profiler PCR arrays, the up-regulation of TNFSF15, IFNA5 and the down-regulation of TLR8 were significant in SB_ODN treated blood samples. A significant up-regulation of TLR3 and a significant down-regulation of TLR9 were detected in CpG_ODN incubated samples. Summarized, this large number of examined genes, totally 26, in RT^2^ Profiler PCR arrays demonstrated mostly similar significant regulations as in the microarray analyses. Thereby, the results of the microarray analyses were successfully validated.

### Functional relevancy of differentially expressed genes

The lists of differentially expressed genes were tested for over-representation in KEGG Pathways and GO terms. The GO and KEGG databases try to arrange genes to specific informative groups. Differentially expressed genes were subjected to a conditional hypergeometric test in the two main branches of GO called “biological process” and “molecular function”. GO categories with a p≤0.01 were considered significantly enriched. Consistently, the over-presentation of differentially expressed genes was examined in known signal transduction and metabolic pathways of KEGG database. The KEGG database provides a sorting of genes depending to which biological pathway they belong. Thus, KEGG Pathway analyses were performed to identify effected pathways by the large number of regulated genes.

In Table 8, list of GO terms are shown for CpG_ODN and SB_ODN incubated blood samples after 4 h. In SB_ODN treated blood samples, the GO term analysis revealed in the branch “biological process” notable over-representation of regulated genes in immune response, inflammatory response, regulation of apoptosis and cell death. Similar GO terms were also obtained for significantly regulated genes in CpG_ODN treated samples. Chemokine activity and cytokine receptor binding dominated the molecular function category in both SB_ODN and CpG_ODN treated samples. List of over-represented GO terms after 2 h incubation with CpG_ODN or SB_ODN can be found in Table S2 in File S1.

**Table 8:** Over-represented Gene Ontology (GO) terms in the list of genes that were significantly regulated in blood samples treated with CpG_ODN or SB_ODN for 4 h compared to untreated samples at p≤0.01.

GOBPID/GOMFID	p value	Odds ratio	Exp. count	Count	Size	Term
*CpG_ODN_4h*						
*Biological Process*						
GO:0006950	<0.001	1.764	257	356	833	response to stress
GO:0006954	<0.001	2.232	90	143	287	inflammatory response
GO:0009607	<0.001	2.267	83	133	266	response to biotic stimulus
GO:0010941	<0.001	1.643	232	310	740	regulation of cell death
GO:0023034	<0.001	1.443	376	463	1199	intracellular signaling pathway
*Molecular Function*						
GO:0005126	<0.001	2.658	38	66	122	cytokine receptor binding
GO:0000287	<0.001	2.056	43	66	138	magnesium ion binding
GO:0005125	<0.001	2.246	32	52	104	cytokine activity
GO:0046983	<0.001	1.876	48	71	157	protein dimerization activity
GO:0042803	<0.001	1.584	89	119	288	protein homodimerization activity

*SB_ODN_4h*						
*Biological Process*						
GO:0006955	<0.001	4.717	6	22	359	immune response
GO:0006954	<0.001	4.425	5	18	287	inflammatory response
GO:0042981	<0.001	2.959	12	30	728	regulation of apoptosis
GO:0010941	<0.001	2.905	12	30	740	regulation of cell death
GO:0006916	<0.001	4.940	3	14	198	anti-apoptosis
*Molecular Function*						
GO:0008009	<0.001	16.938	0	6	28	chemokine activity
GO:0005126	<0.001	5.621	2	11	135	cytokine receptor binding
GO:0005080	0.001	10.675	0	4	27	protein kinase C binding
GO:0001664	0.002	5.011	1	6	80	G-protein-coupled receptor binding
GO:0004871	0.002	1.846	20	33	1208	signal transducer activity

The terms are sorted from highest to lowest significance. A maximum of 5 terms are presented in the list.

GOBPID/GOMFID: Gene Ontology Identifier for Biological Process/Molecular Function; Odds ratio is the ratio of odds that a GO term is enriched in the selected category (extent for the association of GO terms with the differentially expressed genes); Exp. Count represents the expected number of observations in a random selection of x genes; Count represents actual observations; Size is the number of genes on the array that are assigned to the GO term; Term is the short description of the process/function.

KEGG Pathway analyses were performed and affected pathways by the large number of regulated genes in human blood after incubation with CpG_ODN or SB_ODN are shown in Table 9. Because of the small amount of significantly regulated genes after 2 h circulation with SB_ODN, no pathway could be determined for this treatment group. The most significantly enriched gene expression changes both after incubation with SB_ODN and CpG_ODN belonged to the Toll-like receptor signaling pathway. Furthermore, NOD-like receptor signaling pathway, RIG-I-like receptor signaling pathway, cytokine-cytokine receptor interaction, chemokine signaling pathway, and cytosolic DNA-sensing pathway belonged to the top ten of KEGG pathway terms that were significantly affected in SB_ODN as well as CpG_ODN treated blood samples. Other immune response related pathways after incubation with CpG_ODN were apoptosis, lysosome, and regulation of autophagy.

**Table 9:** Over-represented KEGG Pathway terms in the list of genes that were significantly regulated in blood samples treated with CpG_ODN for 2 or 4 h, or SB_ODN for 4 h compared to untreated samples at p≤0.01.

KEGGID	p value	Odds ratio	Exp. count	Count	Size	Term
*CpG_ODN_2h*						
4620	<0.001	3.868	27	56	93	Toll-like receptor signaling pathway
4621	<0.001	5.489	16	39	57	NOD-like receptor signaling pathway
4060	<0.001	2.007	57	87	198	Cytokine-cytokine receptor interaction
4142	<0.001	2.446	32	55	112	Lysosome
4623	<0.001	3.637	14	29	49	Cytosolic DNA-sensing pathway
5120	<0.001	2.866	17	32	60	Epithelial cell signaling in Helicobacter pylori infection
4622	<0.001	2.666	19	34	66	RIG-I-like receptor signaling pathway
4062	<0.001	1.936	43	65	150	Chemokine signaling pathway
4140	<0.001	3.848	8	17	28	Regulation of autophagy
4210	<0.001	2.208	23	37	79	Apoptosis

*CpG_ODN_4h*						
4620	<0.001	4.910	32	66	93	Toll-like receptor signaling pathway
4622	<0.001	6.226	23	50	66	RIG-I-like receptor signaling pathway
4623	<0.001	6.091	17	37	49	Cytosolic DNA-sensing pathway
4621	<0.001	4.650	19	40	57	NOD-like receptor signaling pathway
4210	<0.001	3.234	27	49	79	Apoptosis
4060	<0.001	1.962	68	98	198	Cytokine-cytokine receptor interaction
4142	<0.001	2.284	38	60	112	Lysosome
4140	<0.001	4.883	10	20	28	Regulation of autophagy
4062	<0.001	1.826	51	72	150	Chemokine signaling pathway
5142	<0.001	2.150	31	47	90	Chagas disease

*SB_ODN_4h*						
4620	<0.001	7.227	2	12	93	Toll-like receptor signaling pathway
4621	<0.001	7.635	1	8	57	NOD-like receptor signaling pathway
4622	<0.001	6.435	1	8	66	RIG-I-like receptor signaling pathway
5222	0.001	5.114	2	7	70	Small cell lung cancer
5144	0.001	6.861	1	5	38	Malaria
4060	0.001	3.059	4	12	198	Cytokine-cytokine receptor interaction
4062	0.002	3.343	3	10	150	Chemokine signaling pathway
5142	0.004	3.861	2	7	90	Chagas disease
4623	0.005	5.131	1	5	49	Cytosolic DNA-sensing pathway
5146	0.006	4.068	2	6	73	Amoebiasis

The terms are sorted from highest to lowest significance. A maximum of 10 terms are presented in the list. No pathway could be determined in SB_ODN treated samples for 2 h.

KEGGID: Identifier for Kyoto Encyclopedia of Genes and Genomes Pathway; Odds ratio is the ratio of odds that a KEGG term is enriched in the selected category (extent for the association of KEGG terms with the differentially expressed genes); Exp. Count represents the expected number of observations in a random selection of x genes; Count represents actual observations; Size is the number of genes on the array that are assigned to the KEGG term; Term is the short description of the pathway.

### Functional analysis of regulated transcripts using Ingenuity Pathway Analysis

**Figure 4:**
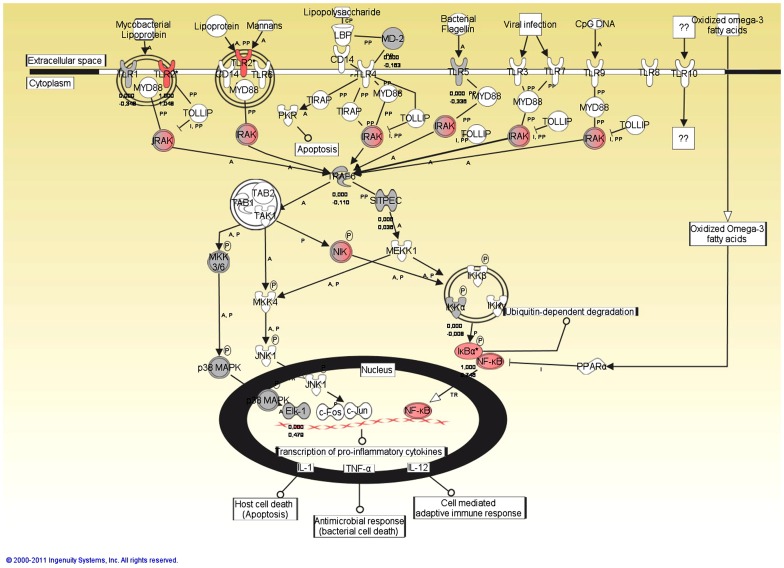
Ingenuity Path Designer representation of Toll-like receptor signaling pathway with affected molecules in SB_ODN treated blood samples for 4 h. Molecules are represented as nodes and the biological relationship between two nodes is represented as an edge (line). All edges are supported by at least one reference from the literature, from a textbook, or from canonical information stored in the Ingenuity Pathways Knowledge Base. The intensity of the node color indicates the degree of up- (red) or down- (green) regulation. Direct relationships are indicated by solid lines. Line beginnings and endings illustrate the direction of the relationship (e.g. arrow head indicates gene A influences gene B). Nodes are displayed using various shapes that represent the functional class of the gene product. Edges are displayed with various labels that describe the nature of the relationship between the nodes (e.g., P for phosphorylation, T for transcription).

For functional analysis of the altered transcripts, gene lists of differentially expressed transcripts were tested for interactions between the regulated genes and possible changes of known signaling and metabolic pathways using the Ingenuity Pathway Analysis (IPA) software. The Ingenuity Path Designer feature was utilized for graphical representation of the molecular relationships between molecules. Since KEGG pathway analyses identified Toll-like receptor pathway as the most affected pathway after SB_ODN stimulation, the regulated molecules involved in this pathway were represented using the Ingenuity Path Designer feature (Figure 4). After stimulation of human blood for 2 h with SB_ODN, there were no significantly regulated transcripts belonging to the TLR signaling pathway, thus Figure 4 shows the affected molecules after 4 h stimulation with SB_ODN. The stimulation of human blood with SB_ODN for 4 h led to an up-regulation of TLR2, interleukin-1 receptor-associated kinase (IRAK), NF-κB inducing kinase (NIK), as well as NF-κB and I-κBα. I-κB kinase (IKK) complex consists of IKK-α, IKK-β, and IKK-γ subunits. IKK phosphorylates the inhibitory I-κBα protein. This phosphorylation leads to the dissociation of I-κBα from NF-κB. NF-κB, which is then free, migrates into the nucleus and activates the expression of pro-inflammatory cytokines that are involved in many important cell processes, including immune response, inflammation, cell death, and cell proliferation.

Figure S1 in File S1 shows Toll-like receptor signaling pathway with affected molecules after circulation of human blood for 4 h with CpG_ODN. The stimulation with CpG_ODN for 4 h affected more molecules than after the stimulation with SB_ODN (Figure 4). Additionally to the up-regulated molecules, which were detected after SB_ODN stimulation, CpG_ODN stimulation led to the up-regulation of MYD88 and protein kinase-R (PKR) and to the down-regulation of amongst others TLR1, 5, 6, 8, and 10. Thereby, TLR5 and 8 were down-regulated more strongly than the other TLRs.

**Figure 5:**
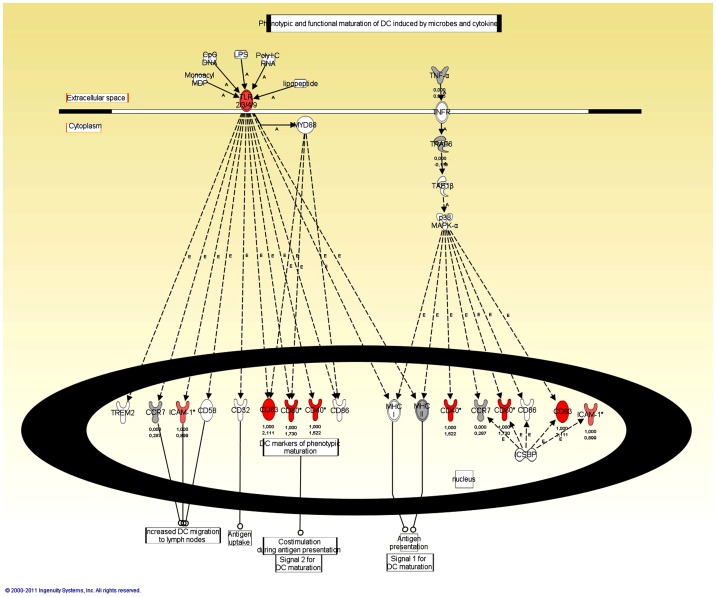
Ingenuity Path Designer representation of dendritic cell maturation with affected molecules in SB_ODN treated blood samples for 4 h. Molecules are represented as nodes, and the biological relationship between two nodes is represented as an edge (line). All edges are supported by at least one reference from the literature, from a textbook, or from canonical information stored in the Ingenuity Pathways Knowledge Base. The intensity of the node color indicates the degree of up- (red) or down- (green) regulation. Direct relationships are indicated by solid lines. Dashed lines denote indirect interactions. Line beginnings and endings illustrate the direction of the relationship (e.g. arrow head indicates gene A influences gene B). Nodes are displayed using various shapes that represent the functional class of the gene product. Edges are displayed with various labels that describe the nature of the relationship between the nodes (e.g., P for phosphorylation, T for transcription).

Furthermore, using Ingenuity Path Designer, we looked more closely to the molecules that are involved in the maturation of dendritic cells. Dentritic cells are one of the most important immune cells, which connect the innate and the acquired immune system. These cells contain TLRs and are able to recognize invading pathogens or components thereof. Therefore, the activation and maturation of dendritic cells can be an additional evidence for immune activation and the disposition of cells to activate T cells. Affected molecules belonging to the maturation of dendritic cells are represented in Figure 5 for samples incubated with SB_ODN for 4 h. Thereby, after a 4-hour incubation with SB_ODN, the maturation markers for human dendritic cells, namely CD83, CD80 (B7–1) and CD40 were up-regulated compared to untreated samples. These marker play an important role in the activation of cellular T cell response. The costimulatory signal, which is necessary to continue the immune response in T cells, comes from B7-CD28 and CD40-CD40L interactions. After 2 h incubation of blood with SB_ODN, only CD83 belonging to the dendritic cell maturation marker was up-regulated (Table 6). Additionally to the maturation markers, ICAM-1 (Intercellular Adhesion Molecule 1) was also up-regulated in SB_ODN treated samples after 4 h, which could be a sign of increased disposition of dendritic cells for migration, for example to the lymph nodes in vivo. After stimulation of blood for 4 h with CpG_ODN (Figure S2 in File S1), same molecules were up-regulated as with SB_ODN incubation for 4 h. However, CD32, which is an antigen uptake receptor on dendritic cells, and MHC-II, which is an activation and maturation marker of dendritic cells, were down-regulated. In contrast, in samples after 2 h stimulation with CpG_ODN, there was an up-regulation of MHC-II (data not shown). Presumably, the up-regulation of MHC-II happens already after 2 h and it is down-regulated after 4 h. Furthermore, the costimulation molecule CD86 (B7–2) was also significantly up-regulated after 2 h incubation with CpG_ODN.

## Discussion

Aptamers are promising ligands for numerous in vivo applications such as for the direct treatment of diseases [21], diagnosis [22], imaging [23], or in vivo tissue engineering [24]. However, for a successful clinical application, aptamers should not lead to an excessive immune activation. According to our literature research, so far there is no study, which examined the immune activation potential of aptamers in human blood. Thus, in this study, the immune activation potential of SB_ODN, which is the starting ssDNA pool for the selection of aptamers, was determined in fresh human blood. CpG_ODN (M362) [25], which belongs to class C CpG ODN with high immune activation potential and is able to activate plasmacytoid dendritic cells as well as B cells that express TLR9 [26], was used as positive control. Using microarray analyses, expression changes were examined in SB_ODN and CpG_ODN treated blood samples after 2 and 4 h circulation in an in vitro closed-loop model.

Endotoxin and pyrogen content of the used ODNs were tested by highly sensitive two independent bioassays, namely by using the LAL assay and monocyte activation test. Detected values were far below the acceptable values. Thereby, undesired effects by possible contaminations of ODNs with endotoxins and pyrogens could be excluded. Furthermore, analyses of blood cell numbers demonstrated that they were not influenced by the circulation in the in vitro closed-loop model and by the addition of both CpG_ODN and SB_ODN. Thereby, the suitability of the test system for immune stimulation studies of single-stranded ODNs in fresh human whole blood was shown.

In comparison to the blood samples without ODN and with SB_ODN, stimulation of human blood with CpG_ODN led to a significantly higher PMN elastase release. Human neutrophils express all known TLRs except TLR3 [27]. Therefore, CpG_ODN could bind to TLR9 in these cells and activate them to secrete PMN elastase to destroy possible bacterial invaders. In contrast, the incubation of human peripheral blood with SB_ODN and CpG_ODN had no influence on plasma concentration of thrombin-antithrombin-III complex (TAT), which was also demonstrated by Paul et al. [28] after 60 minutes incubation of a start library in the in vitro closed-loop model.

Due to the presence of primer regions in the SB_ODN, the stability of unmodified SB_ODN in serum could be examined by quantitative real time PCR analyses. Every 2 hours, the SB_ODN amount in the serum was approximately halved, which was also confirmed by denaturing PAGE electrophoresis. So that after 4-hour incubation, 30% of the initial amount was still present in the serum samples although SB_ODN was used without chemical modifications against degradation. While CpG_ODN contains a complete phosphorothioate backbone which renders them stable against degradation by DNase, denaturing PAGE electrophoresis also showed approximately a halving of CpG_ODN amount every 2 hours. This shows that the increased expression changes in CpG_ODN treated samples compared to SB_ODN treated samples were not caused by the prolonged presence of CpG_ODN in human blood.

Already the treatment of human blood for 2 h with the positive control (CpG_ODN) led to significantly high number of differentially regulated transcripts. In contrast, only after a 4-hour incubation of human blood with SB_ODN in the in vitro closed-loop model, a slightly increased expression change could be detected. Thus, after 2 h of incubation with SB_ODN only 0.03% (10 transcripts) of the examined transcripts and with CpG_ODN 21.19% (6379 transcripts) was regulated. Incubation of human blood with CpG_ODN for 4 h resulted in significant regulation of transcripts, more precisely 25.14% (7570 transcripts) of the examined transcripts. Against it, only 0.98% (295 transcripts) of the examined transcripts was regulated in blood samples incubated with SB_ODN. Microarray analyses clearly showed that the treatment of human blood for 4 h with SB_ODN or CpG_ODN leads to higher percentage of regulated transcripts than after 2 h incubation.

Single-stranded DNA ODNs demonstrated the potential to activate the immune system. Above all, the chemokines CCL8, CXCL10, CCL7, and CXCL11 belonged to the highly up-regulated transcripts after 4 h incubation with SB_ODN. CCL8 is produced by monocytes and shows chemotactic activity to NK cells, monocytes, and T cells. CXCL10 is secreted by several cell types, amongst others, in the human peripheral blood by monocytes. It has like CCL8 a chemotactic activity to monocytes/macrophages, T cells, dendritic cells, and NK cells and promotes the adhesion of T cells to endothelial cells. CCL7 is produced by macrophages and by certain tumor cells, and specifically attracts monocytes and regulates macrophage function. Gene expression of CXCL11 is strongly induced by IFN-β and IFN-γ. It is highly expressed in leukocytes and it is chemotactic for activated T cells. From these regulated chemokines, CCL8, CXCL10, and CCL7 belonged also to the highly up-regulated transcripts in CpG_ODN treated samples after 4 h. However, the expression levels were approximately 15–17 times higher than in SB_ODN treated samples. Also other differentially regulated transcripts in CpG_ODN treated samples showed higher expression levels than after the SB_ODN treatment.

GO terms analyses revealed that the differentially expressed genes after SB_ODN incubation belonged to the transcripts, which are regulated during an immune and inflammatory response. They were also involved in regulation of apoptosis and cell death. Most of regulated transcripts exhibited a chemokine activity. Furthermore, KEGG pathway analyses proved that most of regulated transcripts in SB_ODN as well as CpG_ODN treated samples were overrepresented in the Toll-like receptor signaling pathway. Microarray analyses demonstrated that from all known TLRs, only TLR2 was significantly up-regulated in SB_ODN treated samples after 4 h. In human blood, monocytes express the highest level of TLR2 followed by CD15^+^ granulocytes, CD19^+^ B cells, and CD3^+^ T cells [26], [29]. TLR2 recognizes a variety of microbial products, such as peptidoglycan, lipoteichoic acid, lipoprotein, lipoarabinomannan, and zymosan [30]. Nilsen et al. [31] also demonstrated in their studies an up-regulation of TLR2 on murine macrophages in response to CpG ODN. The up-regulation of TLR2 may lead to enhanced sensitivity of monocytes to microbial components and thereby contribute to a better and fast recognition and elimination of invaders. In addition to TLR signaling pathway, the regulated transcripts in SB_ODN treated samples were overrepresented in NOD-like receptor signaling pathway, retinoic acid inducible gene I (RIG-I)-like receptor signaling pathway, cytokine-cytokine receptor interaction, chemokine signaling pathway, and cytosolic DNA sensing pathway. Cytosolic DNA sensing pathway includes recognition of the cytosolic DNA by DAI, AIM2, and RNA polymerase III, which converts the DNA into RNA for recognition by the RNA sensor RIG-I. DAI is the first identified cytosolic sensor of DNA [10]. It activates the IRF (interferon regulatory factor) and NFκB transcription pathways, which lead to the production of type I interferon and other cytokines. The RNA helicase RIG-I is a cytosolic RNA-binding protein expressed in both immune and nonimmune cells and it does not bind to DNA. However, recent studies confirmed the involvement of RIG-I in recognition of DNA [9] and demonstrated that an RNA intermediate was responsible for the activation of RIG-I [32]. They found that AT-rich dsDNA was transcribed by RNA polymerase III into dsRNA containing a 5′-triphosphate moiety. RIG-I activation by this RNA intermediate induced type I interferon production and activation of the transcription factor NFκB, which is important for example for eliminating viruses. Previous studies have also showed that the NLR (NOD-like receptor) pathway is important for sensing cytosolic DNA and triggering inflammasome dependent innate immune signaling [33]. Inflammasomes are large intracellular multiprotein complexes that lead to the activation of the proteolytic enzyme caspase-1, which promotes the maturation of the proinflammatory cytokines IL-1β and IL-18. Hitherto, four inflammasome complexes have been partially characterized, containing NLRP3, NLRP1, NLRC4 (IPAF), or AIM2. AIM2 recognizes dsDNA [34], [35] and leads to oligomerization of the inflammasome complex by binding to the inflammasome-adaptor protein (ASC, Apoptosis-associated Speck-like protein containing a caspase activation and recruitment domain), which in turn interacts with caspase-1 leading to its activation.

The up-regulation of transcripts CD83, CD80 (B7–1), and CD40 after the incubation of human blood with SB_ODN for 4 hours indicates an activation and maturation of dendritic cells by single-stranded DNA molecules. The next question that arises then is whether these cells are able to activate cells of the acquired immune system. The presence of antibodies against nucleic acids in autoimmune diseases, such as systemic lupus erythematosus, suggests the possibility of the acquired immune system activation by ssDNA molecules. Recently, Karbarch and colleagues [36] demonstrated the formation of anti-CpG antibodies after the therapeutic administration of a synthetic CpG ODN. These results prove the potential of ODNs to stimulate human B cells to produce specific antibodies. Therefore, a possible activation of the adaptive immune system by ssDNA oligonucleotides (aptamers) remains to be determined in more detail.

26 genes were selected for the validation of microarray data by RT^2^ Profiler PCR arrays. Expression of investigated genes demonstrated for the most part accordance with the determined transcript regulations in microarray analyses. However, the determined fold changes in RT^2^ Profiler PCR arrays were continuously higher than the determined fold changes in microarray analyses. Microarray chips and qRT-PCR use different detection methods to determine differentially expressed genes. Higher fold change values in the RT^2^ Profiler PCR arrays indicate a higher sensitivity of this detection method compared to microarray analyses.

The used SB_ODN is a combinatorial ssDNA library consisting of approximately 10^15^ different ssDNA molecules. It is possible that only a small amount of ssDNA molecules with certain sequences were recognized by PRRs, such as TLR9. Therefore, the application of purified potentially TLR9 recognizing ssDNA molecules in higher concentrations can lead to a higher activation of the immune system than measured in this study after the incubation of human blood with SB_ODN. These ssDNA molecules can contain TLR9 binding and activating CpG-motifs. However, additionally to the CpG motif containing DNA, several non-CpG motif containing nucleic acid TLR9 ligands [37]–[39] have been identified with the ability to activate TLR9. This shows that TLR9 has a much wider range of specificity than only CpG-motifs. Single-stranded DNA can fold into three-dimensional structures and can bind with high affinity to different targets. The fact that already TLR9 binding aptamers exist, shows that TLR9 can serve as a potential target for ssDNA molecules [40], [41]. Therefore, aptamers for in vivo applications should be previously tested in vitro for their immune activation potential.

The in vivo application of aptamers is a promising approach in regenerative medicine, imaging, cancer diagnosis, hemostatic control, or treatment of several diseases. In this study, immune activation potential of aptamers in human blood was examined using microarray analyses. Although transcriptional changes were significantly less than in CpG_ODN treated samples, after 4 h of ssDNA ODN incubation, a differential regulation of transcripts belonging to the inflammatory and immune response was determined. Thus, we highly recommend performing of these preclinical tests prior to clinical application of new aptamer-based therapies.

## Supporting Information

File S1 Table S1: Validation of microarray data using RT^2^ Profiler PCR Array. Comparative list of determined expression changes of selected transcripts after 2 h of blood incubation with SB_ODN or CpG_ODN using RT^2^ Profiler PCR Array and the microarray analysis. Table S2: Over-represented Gene Ontology (GO) terms in the list of genes that were significantly regulated in blood samples treated with CpG_ODN or SB_ODN for 2 h compared to untreated samples at p≤0.01. The terms are sorted from highest to lowest significance. A maximum of 5 terms are presented in the list. Figure S1: Ingenuity Path Designer representation of Toll-like receptor signaling pathway with affected molecules in CpG_ODN treated blood samples for 4 h. Molecules are represented as nodes, and the biological relationship between two nodes is represented as an edge (line). All edges are supported by at least one reference from the literature, from a textbook, or from canonical information stored in the Ingenuity Pathways Knowledge Base. The intensity of the node color indicates the degree of up- (red) or down- (green) regulation. Direct relationships are indicated by solid lines. Line beginnings and endings illustrate the direction of the relationship (e.g. arrow head indicates gene A influences gene B). Nodes are displayed using various shapes that represent the functional class of the gene product. Edges are displayed with various labels that describe the nature of the relationship between the nodes (e.g., P for phosphorylation, T for transcription). Figure S2: Ingenuity Path Designer representation of dendritic cell maturation with affected molecules in CpG_ODN treated blood samples for 4 h. Molecules are represented as nodes, and the biological relationship between two nodes is represented as an edge (line). All edges are supported by at least one reference from the literature, from a textbook, or from canonical information stored in the Ingenuity Pathways Knowledge Base. The intensity of the node color indicates the degree of up- (red) or down- (green) regulation. Direct relationships are indicated by solid lines. Dashed lines denote indirect interactions. Line beginnings and endings illustrate the direction of the relationship (e.g. arrow head indicates gene A influences gene B). Nodes are displayed using various shapes that represent the functional class of the gene product. Edges are displayed with various labels that describe the nature of the relationship between the nodes (e.g., P for phosphorylation, T for transcription).
(DOCX)
