# Supplementary material for: Potential Capacity of Aptamers to Trigger Immune Activation in Human Blood
Source: PLoS One. 2013 Jul 23;8(7):e68810. doi: 10.1371/journal.pone.0068810 (PMC3720859; doi:10.1371/journal.pone.0068810)
Supplement: File S1 — Table S1: Validation of microarray data using RT2 Profiler PCR Array. Comparative list of determined expression changes of selected transcripts after 2 h of blood incubation with SB_ODN or CpG_ODN using RT2 Profiler PCR Array and the microarray analysis. Table S2: Over-represented Gene Ontology (GO) terms in the list of genes that were significantly regulated in blood samples treated with CpG_ODN or SB_ODN for 2 h compared to untreated samples at p≤0.01. The terms are sorted from highest to lowest significance. A maximum of 5 terms are presented in the list. Figure S1: Ingenuity Path Designer representation of Toll-like receptor signaling pathway with affected molecules in CpG_ODN treated blood samples for 4 h. Molecules are represented as nodes, and the biological relationship between two nodes is represented as an edge (line). All edges are supported by at least one reference from the literature, from a textbook, or from canonical information stored in the Ingenuity Pathways Knowledge Base. The intensity of the node color indicates the degree of up- (red) or down- (green) regulation. Direct relationships are indicated by solid lines. Line beginnings and endings illustrate the direction of the relationship (e.g. arrow head indicates gene A influences gene B). Nodes are displayed using various shapes that represent the functional class of the gene product. Edges are displayed with various labels that describe the nature of the relationship between the nodes (e.g., P for phosphorylation, T for transcription). Figure S2: Ingenuity Path Designer representation of dendritic cell maturation with affected molecules in CpG_ODN treated blood samples for 4 h. Molecules are represented as nodes, and the biological relationship between two nodes is represented as an edge (line). All edges are supported by at least one reference from the literature, from a textbook, or from canonical information stored in the Ingenuity Pathways Knowledge Base. The intensity of the node color in [file pone.0068810.s001.docx]

**SUPPLEMENTARY INFORMATION**

**Table S1**

|  |  | **qRT-PCR** | | **Microarray Analyses** | |
| --- | --- | --- | --- | --- | --- |
| **Gene** | **Treatment** | **p-Value** | **Up- or Down-Regulation** | **M** | **Fold Change** |
| **CXCL10** | CpG_ODN_2h | **<0.01** | **85.76** | **4.75** | **26.82** |
|  | SB_ODN_2h | 0.245 | 1.20 | 0.26 | 1.20 |
| **CCL7** | CpG_ODN_2h | **<0.01** | **806.51** | **7.25** | **151.76** |
|  | SB_ODN_2h | 0.086 | 1.94 | 0.97 | 1.95 |
| **CCL2** | CpG_ODN_2h | **<0.01** | **675.59** | **7.52** | **183.81** |
|  | SB_ODN_2h | 0.078 | 2.21 | 1.16 | 2.23 |
| **IL6** | CpG_ODN_2h | **<0.01** | **94.79** | **4.27** | **19.31** |
|  | SB_ODN_2h | **<0.01** | **4.19** | 0.45 | 1.36 |
| **CXCL11** | CpG_ODN_2h | **<0.01** | **14.36** | 0.61 | 1.53 |
|  | SB_ODN_2h | 0.403 | 1.19 | -0.15 | -1.11 |
| **TLR2** | CpG_ODN_2h | **<0.01** | **3.35** | **2.24** | **4.72** |
|  | SB_ODN_2h | 0.427 | -1.09 | 0.33 | 1.26 |
| **ICAM1** | CpG_ODN_2h | **<0.01** | **3.32** | **2.06** | **4.16** |
|  | SB_ODN_2h | 0.362 | 1.06 | 0.47 | 1.38 |
| **PLAU** | CpG_ODN_2h | **<0.01** | **348.36** | **7.70** | **208.08** |
|  | SB_ODN_2h | 0.106 | 1.59 | 0.95 | 1.93 |
| **SERPINB2** | CpG_ODN_2h | **<0.01** | **118.51** | **5.91** | **60.22** |
|  | SB_ODN_2h | 0.428 | -1.03 | 0.33 | 1.26 |
| **CD83** | CpG_ODN_2h | **<0.01** | **41.58** | **4.93** | **30.39** |
|  | SB_ODN_2h | **< 0.05** | **2.64** | **1.72** | **3.30** |
| **CD40** | CpG_ODN_2h | **<0.01** | **5.74** | **2.41** | **5.32** |
|  | SB_ODN_2h | **< 0.05** | **1.31** | 0.53 | 1.44 |
| **CD80** | CpG_ODN_2h | **<0.01** | **14.15** | **2.56** | **5.91** |
|  | SB_ODN_2h | **< 0.05** | **1.80** | 0.20 | 1.15 |
| **IL4I1** | CpG_ODN_2h | **<0.01** | **12.03** | **3.42** | **10.67** |
|  | SB_ODN_2h | 0.167 | 1.32 | 0.70 | 1.62 |
| **TNFSF15** | CpG_ODN_2h | **<0.01** | **310.36** | **5.95** | **61.92** |
|  | SB_ODN_2h | 0.058 | 2.11 | 0.18 | 1.13 |
| **IFNA5** | CpG_ODN_2h | **<0.01** | **64.00** | **5.50** | **45.23** |
|  | SB_ODN_2h | 0.169 | -1.37 | -0.14 | -1.10 |
| **IFNB1** | CpG_ODN_2h | **<0.01** | **178.25** | **5.53** | **46.27** |
|  | SB_ODN_2h | 0.342 | -1.15 | 0.04 | 1.03 |
| **RGL1** | CpG_ODN_2h | **<0.01** | **8.57** | **3.23** | **9.38** |
|  | SB_ODN_2h | 0.263 | 1.04 | 0.21 | 1.17 |
| **CD22** | CpG_ODN_2h | **<0.01** | **4.39** | **1.85** | **3.59** |
|  | SB_ODN_2h | 0.291 | 1.14 | 0.62 | 1.54 |
| **PTPN7** | CpG_ODN_2h | **<0.01** | **2.00** | **1.16** | **2.24** |
|  | SB_ODN_2h | 0.211 | 1.24 | **0.62** | **1.53** |
| **F2RL1** | CpG_ODN_2h | **<0.01** | **-21.11** | **-3.30** | **-9.85** |
|  | SB_ODN_2h | **< 0.05** | **-1.87** | -0.47 | -1.39 |
| **TLR1** | CpG_ODN_2h | **<0.01** | **-10.64** | **-2.64** | **-6.23** |
|  | SB_ODN_2h | 0.271 | -1.52 | -0.17 | -1.12 |
| **TLR5** | CpG_ODN_2h | **<0.01** | **-4.22** | **-2.62** | **-6.15** |
|  | SB_ODN_2h | 0.156 | -1.46 | -0.25 | -1.19 |
| **TLR8** | CpG_ODN_2h | **<0.01** | **-7.58** | **-2.61** | **-6.10** |
|  | SB_ODN_2h | **<0.01** | **-1.54** | -0.22 | -1.17 |
| **TLR9** | CpG_ODN_2h | **<0.01** | **-1.98** | n.d. | n.d. |
|  | SB_ODN_2h | 0.975 | -1.02 | n.d. | n.d. |
| **TLR3** | CpG_ODN_2h | 0.346 | -1.22 | n.d. | n.d. |
|  | SB_ODN_2h | **< 0.05** | **-1.54** | n.d. | n.d. |
| **TLR7** | CpG_ODN_2h | **<0.01** | **-1.64** | **-0.94** | **-1.92** |
|  | SB_ODN_2h | 0.145 | -1.20 | n.d. | n.d. |
| Values, which are significantly up- or down-regulated are written in bold. Not significantly regulated values are not highlighted. The abbreviation n.d. means not detected. | | | | | |

**Table S2**

| GOBPID/GOMFID | p value | Odds ratio | Exp. count | | Count | | Size | | Term | | |
| --- | --- | --- | --- | --- | --- | --- | --- | --- | --- | --- | --- |
| *CpG_ODN_2h* |  |  |  | |  | |  | |  | | |
| *Biological Process* |  |  |  | |  | |  | |  | | |
| GO:0042981 | < 0.001 | 1.599 | | 199 | | 267 | | 728 | | [regulation of apoptosis](http://www.godatabase.org/cgi-bin/amigo/go.cgi?view=details&search_constraint=terms&depth=0&query=GO:0042981) | |
| GO:0010941 | < 0.001 | 1.576 | | 202 | | 269 | | 740 | | [regulation of cell death](http://www.godatabase.org/cgi-bin/amigo/go.cgi?view=details&search_constraint=terms&depth=0&query=GO:0010941) | |
| GO:0006954 | < 0.001 | 1.983 | | 78 | | 121 | | 287 | | [inflammatory response](http://www.godatabase.org/cgi-bin/amigo/go.cgi?view=details&search_constraint=terms&depth=0&query=GO:0006954) | |
| GO:0023034 | < 0.001 | 1.438 | | 327 | | 408 | | 1199 | | [intracellular signaling pathway](http://www.godatabase.org/cgi-bin/amigo/go.cgi?view=details&search_constraint=terms&depth=0&query=GO:0023034) | |
| GO:0016265 | < 0.001 | 1.457 | | 277 | | 349 | | 1013 | | [death](http://www.godatabase.org/cgi-bin/amigo/go.cgi?view=details&search_constraint=terms&depth=0&query=GO:0016265) | |
| *Molecular Function* |  |  | |  | |  | |  | |  | |
| GO:0005126 | < 0.001 | 2.389 | | 35 | | 60 | | 129 | | [cytokine receptor binding](http://www.godatabase.org/cgi-bin/amigo/go.cgi?view=details&search_constraint=terms&depth=0&query=GO:0005126) | |
| GO:0005125 | < 0.001 | 2.177 | | 28 | | 46 | | 104 | | [cytokine activity](http://www.godatabase.org/cgi-bin/amigo/go.cgi?view=details&search_constraint=terms&depth=0&query=GO:0005125) | |
| GO:0005515 | < 0.001 | 1.184 | | 1326 | | 1404 | | 5045 | | [protein binding](http://www.godatabase.org/cgi-bin/amigo/go.cgi?view=details&search_constraint=terms&depth=0&query=GO:0005515) | |
| GO:0030145 | 0.001 | 3.559 | | 8 | | 17 | | 30 | | [manganese ion binding](http://www.godatabase.org/cgi-bin/amigo/go.cgi?view=details&search_constraint=terms&depth=0&query=GO:0030145) | |
| GO:0019003 | 0.001 | 4.231 | | 6 | | 14 | | 23 | | [GDP binding](http://www.godatabase.org/cgi-bin/amigo/go.cgi?view=details&search_constraint=terms&depth=0&query=GO:0019003) | |
| *SB_ODN_2h* |  |  | |  | |  | |  | |  | |
| *Biological Process* |  |  | |  | |  | |  | |  | |
| GO:0045580 | < 0.001 | 111.511 | | 0 | | 2 | | 47 | | [regulation of T cell differentiation](http://www.godatabase.org/cgi-bin/amigo/go.cgi?view=details&search_constraint=terms&depth=0&query=GO:0045580) | |
| GO:0033085 | 0.001 | 2016.000 | | 0 | | 1 | | 2 | | [negative regulation of T cell differentiation in the thymus](http://www.godatabase.org/cgi-bin/amigo/go.cgi?view=details&search_constraint=terms&depth=0&query=GO:0033085) | |
| GO:0070245 | 0.002 | 1007.900 | | 0 | | 1 | | 3 | | [positive regulation of thymocyte apoptosis](http://www.godatabase.org/cgi-bin/amigo/go.cgi?view=details&search_constraint=terms&depth=0&query=GO:0070245) | |
| GO:0032713 | 0.002 | 671.867 | | 0 | | 1 | | 4 | | [negative regulation of interleukin-4 production](http://www.godatabase.org/cgi-bin/amigo/go.cgi?view=details&search_constraint=terms&depth=0&query=GO:0032713) | |
| GO:0051249 | 0.003 | 35.248 | | 0 | | 2 | | 143 | | [regulation of lymphocyte activation](http://www.godatabase.org/cgi-bin/amigo/go.cgi?view=details&search_constraint=terms&depth=0&query=GO:0051249) | |
| *Molecular Function* |  |  | |  | |  | |  | |  | |
| GO:0004198 | 0.003 | 450.292 | | 0 | | 1 | | 9 | | [calcium-dependent cysteine-type endopeptidase activity](http://www.godatabase.org/cgi-bin/amigo/go.cgi?view=details&search_constraint=terms&depth=0&query=GO:0004198) | |
| GO:0051059 | 0.006 | 240.000 | | 0 | | 1 | | 16 | | [NF-kappaB binding](http://www.godatabase.org/cgi-bin/amigo/go.cgi?view=details&search_constraint=terms&depth=0&query=GO:0051059) | |
| GOBPID/GOMFID: Gene Ontology Identifier for Biological Process/Molecular Function; Odds ratio is the ratio of odds that a GO term is enriched in the selected category (extent for the association of GO terms with the differentially expressed genes); Exp. Count represents the expected number of observations in a random selection of x genes; Count represents actual observations; Size is the number of genes on the array that are assigned to the GO term; Term is the short description of the process/function. | | | | | | | | | | |  |

**
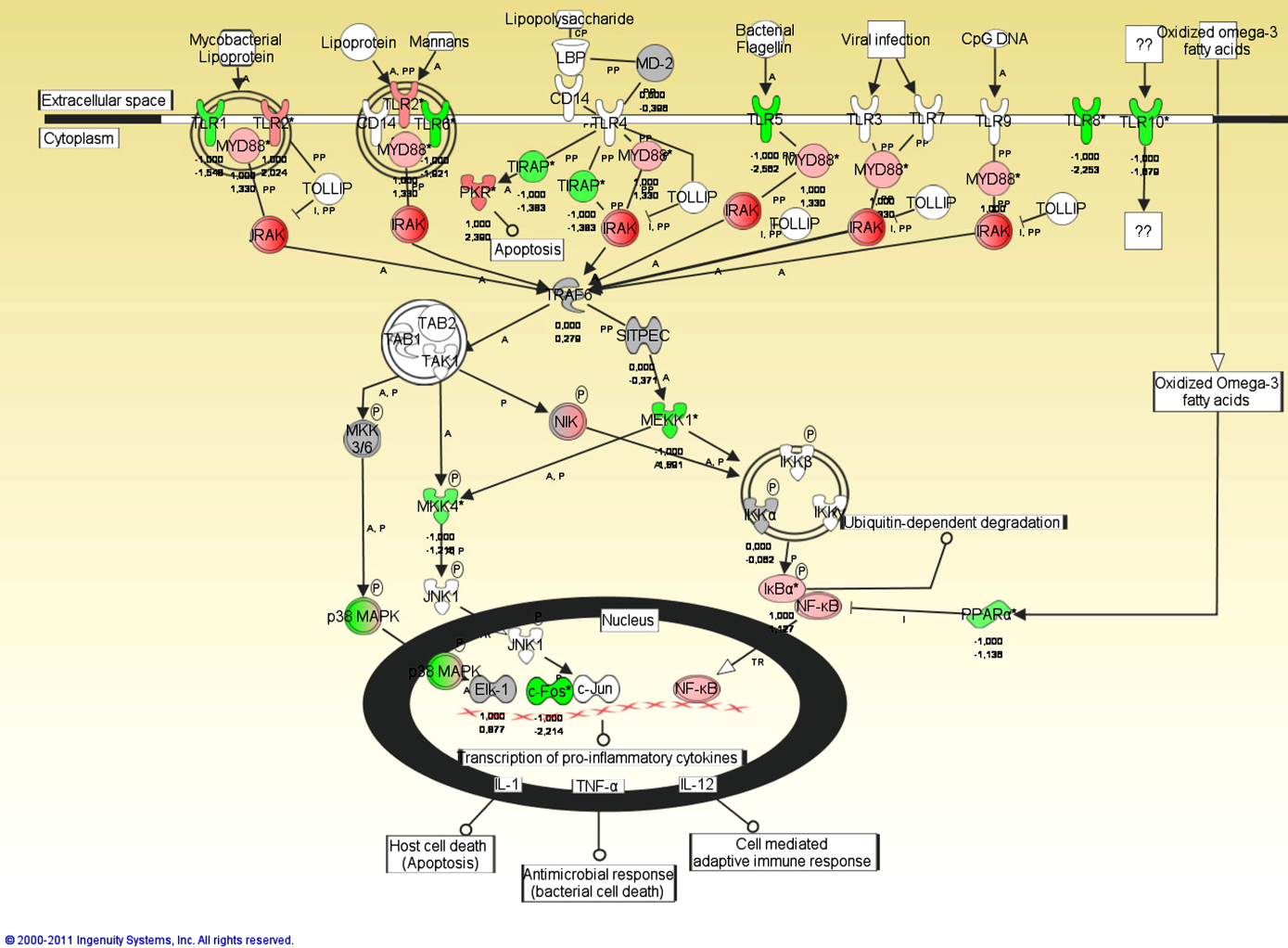
**

**Figure S1**

**
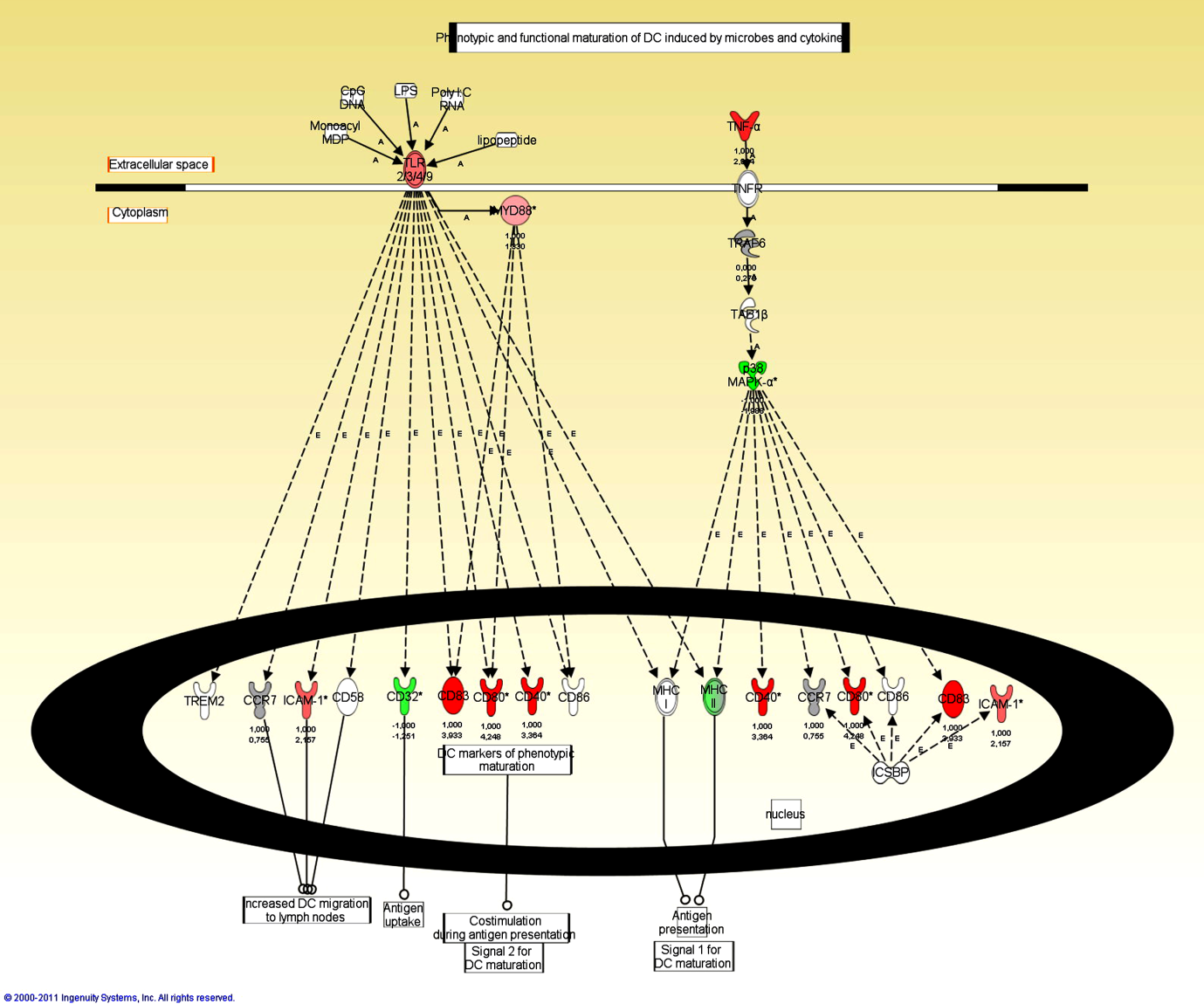
**

**Figure S2**
